# Hypofractionated radiotherapy with simultaneous integrated boost for localized prostate cancer patients: effects on immune system and prediction of toxicity

**DOI:** 10.3389/fimmu.2024.1457839

**Published:** 2024-10-28

**Authors:** Fiorella D’Auria, Luciana Valvano, Giovanni Calice, Vittoria D’Esposito, Serena Cabaro, Pietro Formisano, Gabriella Bianchino, Antonio Traficante, Antonella Bianculli, Grazia Lazzari, Teodora Statuto, Luciana Rago

**Affiliations:** ^1^ Laboratory of Clinical Pathology, Centro di Riferimento Oncologico della Basilicata (IRCCS-CROB), Rionero in Vulture, Italy; ^2^ Laboratory of Clinical Research and Advanced Diagnostics, Centro di Riferimento Oncologico della Basilicata (IRCCS-CROB), Rionero in Vulture, Italy; ^3^ Laboratory of Preclinical and Translational Research, Centro di Riferimento Oncologico della Basilicata (IRCCS-CROB), Rionero in Vulture, Italy; ^4^ Università degli Studi di Napoli “Federico II”, Department of Translational Medical Sciences, Napoli, Italy; ^5^ Radiotherapy Unit, Centro di Riferimento Oncologico della Basilicata (IRCCS-CROB), Rionero in Vulture, Italy

**Keywords:** radiotherapy, prostate cancer, hypofractionation, toxicity, immune cells, cytokines

## Abstract

**Background:**

The other side of radiotherapy (RT), in addition to the cytotoxic effect, is the ability to modulate the immune system in terms of activation or suppression, also depending on the dose and fractionation delivered. This immune RT effect can be detected both locally in the irradiated tumor site and in the peripheral blood. The aim of this study was to assess the consequence of pelvic irradiation on peripheral immune cells and cytokine secretions in localized prostate cancer (PC) patients undergoing pelvic irradiation with a simultaneous moderately hypofractionated prostate/prostate bed boost by Volumetric Modulated Arc Therapy (VMAT). Furthermore, we analyzed whether there was a correlation between these peripheral immune parameters and acute and late genitourinary (GU) and gastrointestinal (GI) toxicity.

**Methods:**

Thirty-eight PC patients were treated with pelvis irradiation (dose per fraction 1.8 Gy) and simultaneous hypofractionated (median dose per fraction: 2.7 Gy) prostate/prostate bed boost. A longitudinal analysis was performed for 12 months on peripheral blood to assess changes in 9 different lymphocyte subpopulations by flow cytometry and 10 circulating cytokines by Multiplex Luminex assay and ELISA.

**Results:**

Our analysis revealed that basal IFN-γ serum values were significantly lower in the definitive (curative intent for patients with prostate) patient group respect to the post-operative one. All the lymphocyte subsets and IFN-α, IFN-β and Il-2 peripheral concentrations displayed significant variations between the different time points considered. The immune cell population that suffers the greatest RT toxicity in the blood was B lymphocyte. We found an interesting correlation between basal TGF-β1 and late GU toxicity. In particular, TGF-β1 concentrations before RT were significantly higher in patients that experienced grade 2-3 of late GU toxicity, respect to grade 0-1. Exploring possible correlations between some clinical/biological findings and radiation planning parameters, we found no statistical significance.

**Conclusions:**

Our study analyzed, in the context of hypofractionated radiotherapy in prostate cancer, different parameters of the peripheral immune system. We have highlighted longitudinally the peripheral behavior of the different lymphocyte subpopulations and of a group of 10 cytokines during the first year after RT. One of the analyzed cytokines, such as TGF-β1, has proven to be promising predictive factor of severe late GU toxicity.

## Introduction

Radiotherapy (RT) is one of the anti-tumor therapeutic strategies in different types of cancers, thanks to its property of killing neoplastic cells by irreparable damage induced to DNA. In addition to the classical role of RT, recently, its possible ability to modulate the immune system’s response against tumors has also been much studied (1–11). The local immunomodulatory RT effects can be immune-stimulatory but, in contrast, also immune-suppressive, also based on the dose and fractionation delivered (7, 12). As regards the circulating immune cells, lymphocytes (particularly B cells) are more sensitive to radiation respect to myeloid cells (7).

With these premises, preclinical (13–15) and clinical studies (16, 17) on the possible combination of the best RT dose and fraction with immunotherapy have emerged.

Studying how different RT doses and schedules can act on the patient immune system is important to be able to design therapeutic combinations of RT with immunomodulatory agents in the future.

Although improved modern radiotherapy techniques target tumor tissues more precisely, normal cells in the surrounding tissues can still be damaged (18). The RT effects on the immune system can be different: induction of immunogenic cell death, activation of dendritic cells, reduction of Treg cells in the tumor microenvironment, increased expression of tumor-associated antigens, activation of tumor-specific T lymphocytes, increased production of pro-inflammatory cytokines (5, 19). RT-induced acute phase of inflammation, which in some patients does not resolve and results in a state of chronic inflammation, may contribute to the development of fibrosis in normal tissues surrounding the tumor and therefore of late toxicity (7, 20, 21).

With these assumptions, the aim of our work was to assess the consequence of hypofractionated RT on prostate/prostate bed with concomitant prophylactic irradiation of the pelvis, on peripheral immune cells and cytokine secretions in localized prostate cancer patients. Furthermore, we looked, among the components of the immune system studied, which could be a predictive factor for RT toxicity, since it is an important aspect for clinicians.

## Methods

### Patients’ characteristics

Thirty-eight prostate cancer patients were prospectively enrolled in our single-institutional study between April 2020 and November 2021. The study was examined and approved by the local ethics committee (Comitato Etico Unico Regionale per la Basilicata, approval number 20170016087) and therefore conducted in accordance with the ethical principles of the Declaration of Helsinki. All enrolled patients were treated according to our Institutional guidelines. In particular, 17 patients underwent definitive moderately hypofractionated RT and 21 patients underwent post-operative moderately hypofractionated RT. **Table 1** reports the details of the doses and fractionations used for the two groups of patients. Hormone therapy was prescribed to 26 patients (**Table 2**). Median duration time of the hormone therapy was 24 months for both groups of patients (definitive range: 12-54 months; post-operative range: 6–36 months).

**Table 1:** Doses and fractionations.

	DEFINITIVE RT (*n*= 17)	POST-OPERATIVE RT (*n*= 21)
Total RT dose Gy, median (range)	67.5 (66.25–68.75)	67.5 (66.25–70)
Number of fractions, median (range)	25 (25–27)	27 (25–28)
Dose per fraction Gy, median (range)	2.7 (2.5–2.75)	2.5 (2.5–2.7)
EQD2 Gy (α/β: 1.5), median (range)	81 (77.14–83.48)	80 (77.14–81)
Pelvis RT dose Gy, median (range)	45 (45–48.6)	48.6 (45–50.4)
Pelvis dose per fraction Gy	1.8	1.8

RT, radiotherapy; EQD2, equivalent dose in 2Gy fractions.

**Table 2:** Patient characteristics.

	ALL PATIENTS (*n*= 38)		DEFINITIVE RT (*n*= 17)		POST-OPERATIVE RT (*n*= 21)	
Variable	Number of patients or median	Percentage or IQR	Number of patients or median	Percentage or IQR	Number of patients or median	Percentage or IQR
Age (years)	73	69-79	79	75-82	71	67-73
Hormone therapy	26	68.4%	15	88.2%	11	52.4%
Gleason Grade Group						
1	5	13.2%	2	11.8%	3	14.3%
2-3	17	44.7%	6	35.3%	11	52.4%
4-5	16	42.1%	9	52.9%	7	33.3%
Clinical T stage						
cT2	14 (a:1; b:9; c:4)	36.8%	14 (a:1; b:9; c:4)	82.4%		
cT3	3 (a:2; b:1)	7.9%	3 (a:2; b:1)	17.6%		
Pathological T stage						
pT2	6 (a:1; b:2; c:3)	15.8%			6 (a:1; b:2; c:3)	28.6%
pT3	15 (a:7; b:8)	39.5%			15 (a:7; b:8)	71.4%
AJCC Stage						
IIA	2	5.3%	0	0%	2	9.5%
IIB	1	2.6%	0	0%	1	4.8%
IIC	12	31.6%	10	58.8%	2	9.5%
IIIA	4	10.5%	3	17.65%	1	4.8%
IIIB	12	31.6%	3	17.65%	9	42.85%
IIIC	3	7.9%	1	5.9%	2	9.5%
IVA	4	10.5%	0	0%	4	19.1%
Margins at prostatectomy (R1)	10	26.3%			10	47.6%
Biochemical relapse	3	7.9%	0	0%	3	14.3%

IQR, interquartile range; AJCC, American Joint Committee on Cancer.

Target volumes were: prostate + seminal vesicles + pelvis for definitive RT; prostate bed + pelvis for post-operative RT. For both RT treatments, patients received a daily dose of 1.8 Gy to the pelvis and a simultaneous integrated boost (SIB), with median dose per fraction of 2.7 Gy (2.5-2.75), to the prostate/prostate bed. For the cranial limit of the pelvis, the right and left common iliac lymph nodes were included. Both groups were treated by Volumetric Modulated Arc therapy (VMAT).

Peripheral blood was collected before RT (t0), at the end of RT (t1), at follow-up time of 3 (t2), 6 (t3) and 12 months (t4) after the end of RT, respectively for 37, 38, 34, 33 and 28 patients.

At the above mentioned time points, absolute counts of white blood cells (WBC) and lymphocytes (ALC) were determined with a Beckman Coulter DXH800.

The genitourinary (GU) and gastrointestinal (GI) RT toxicity of the patients was recorded taking into account the Common Terminology Criteria for Adverse Events (CTCAE) version 5.0. For acute toxicity, we considered the events that occurred in the 120 days following the start of RT. The patients were subjected to the IPSS (International Prostate Symptom Score) questionnaire (22) for an objective evaluation of their urinary symptoms due to the prostatic disease.

### Flow cytometry

For flow cytometry analysis, fluorescence-labeled antibodies were mixed with 100 µl of peripheral blood and incubated for 15 min in the dark. The antibody combinations were CD3-FITC/CD16CD56-PE/CD45-PerCP/CD8-PE-Cy7/CD4-APC/CD19-APC-Cy7; CD127-PE/CD4-PerCP/CD25-PE-Cy7/CD45-APC-Cy7. For each combination, 50,000 to 100,000 CD45-positive cells were analyzed. The peripheral blood lymphocyte subpopulations considered for this study are previously described (8, 23). The gating strategy is shown in **Supplementary Figures 1** and **2**. The absolute number of each cell subpopulation was obtained using the percentage and the ALC values.

### Cytokines

Serum samples were tested to quantify 9 human cytokines (IFN-α, IFN-β, IFN-γ, IL-1β, IL-2, IL-6, IL-8, IL-10, TNF-α) with Multiplex Luminex assay kit (R&D Systems) on Luminex 200 System and TGF-β1 by ELISA (R&D Systems).

### Statistical analysis

We have checked the normal distribution of all variable values by Shapiro–Wilk’s normality method. Since not all data had a normal course, we chose to represent them by median and interquartile range (IQR) and to apply the non-parametric statistical Kruskal–Wallis test and Wilcoxon rank sum test. Correlation was evaluated by Spearman’s rank method. Statistical significance was established on p-value<0.05. All the statistical analyses were performed by R software and CRAN packages (24); customized images were processed by the ggpubr package (25).

## Results

Patient characteristics are reported in **Table 2**. Baseline data of cell population and cytokine concentrations are summarized in **Table 3**. With a median follow-up of 36 months (range 24-50), three patients (7.9%) experienced biochemical relapse, all in the post-operative RT setting. Only one patient (2.6%) died due to a second neoplasm.

**Table 3:** Cell populations and cytokines values at baseline.

Parameter	Median, Range
WBC	6685 (3830-12370) µl^-1^
ALC	1850 (800-3000) µl^-1^
B cells	127.6 (21.6-275) µl^-1^
NK cells	332.5 (108-1043.7) µl^-1^
Total T cells	1180.8 (363.2-1915) µl^-1^
CD4+ T cells	779 (238.4-1487.5) µl^-1^
CD8+ T cells	326.4 (22.35-727.7) µl^-1^
Tregs	42.2 (10.3-103.5) µl^-1^
DNT cells	24.7 (8-87.5) µl^-1^
DPT cells	7.05 (1.6-60) µl^-1^
NKT cells	66.5 (4-275.2) µl^-1^
IFN-α	1.51 (0.25-6.45) pg/ml
IFN-γ	13.87 (9.03-46.69) pg/ml
IL-2	7.68 (5.45-85.83) pg/ml
IL-8	14.97 (5.23-9829.89) pg/ml
TNF-α	4.43 (1.61-115.73) pg/ml
IFN-β	2.80 (0-23.62) pg/ml
IL-1β	0 (0-6210.53) pg/ml
IL-6	0.50 (0-1551.03) pg/ml
IL-10	0.90 (0.10-257.24) pg/ml
TGF-β1	2420 (1147-3693) pg/ml

WBC, white blood cell; ALC, absolute lymphocyte count; NK, natural killer; Tregs, regulatory T cells; DNT, double-negative T; DPT, double-positive T; IFN, interferon; IL, interleukin; TNF, tumor necrosis factor; TGF, transforming growth factor.

### Correlation of baseline values ​​with patient characteristics

We analyzed whether the baseline values ​​of the different cell types and cytokines were influenced by: age, Gleason score, AJCC stage, TNM group, and type of RT (definitive or post-operative). We found that the only parameter significantly correlated with age was DNT value (p-value: 0.014, rho: -0.399). As regards the treatment, basal serum concentration of IFN-γ was significantly lower in definitive patients group (median: 12.7 pg/ml; IQR: 12.1-14.3 pg/ml) respect to post-operative one (median: 13.9 pg/ml; IQR: 13.9-18.7 pg/ml) (p-value: 0.039). We did not find any other influence of the variables considered on the values ​​of the lymphocyte subtypes and cytokines.

### Changes in lymphocyte subpopulations and cytokines over time

**Figure 1:**
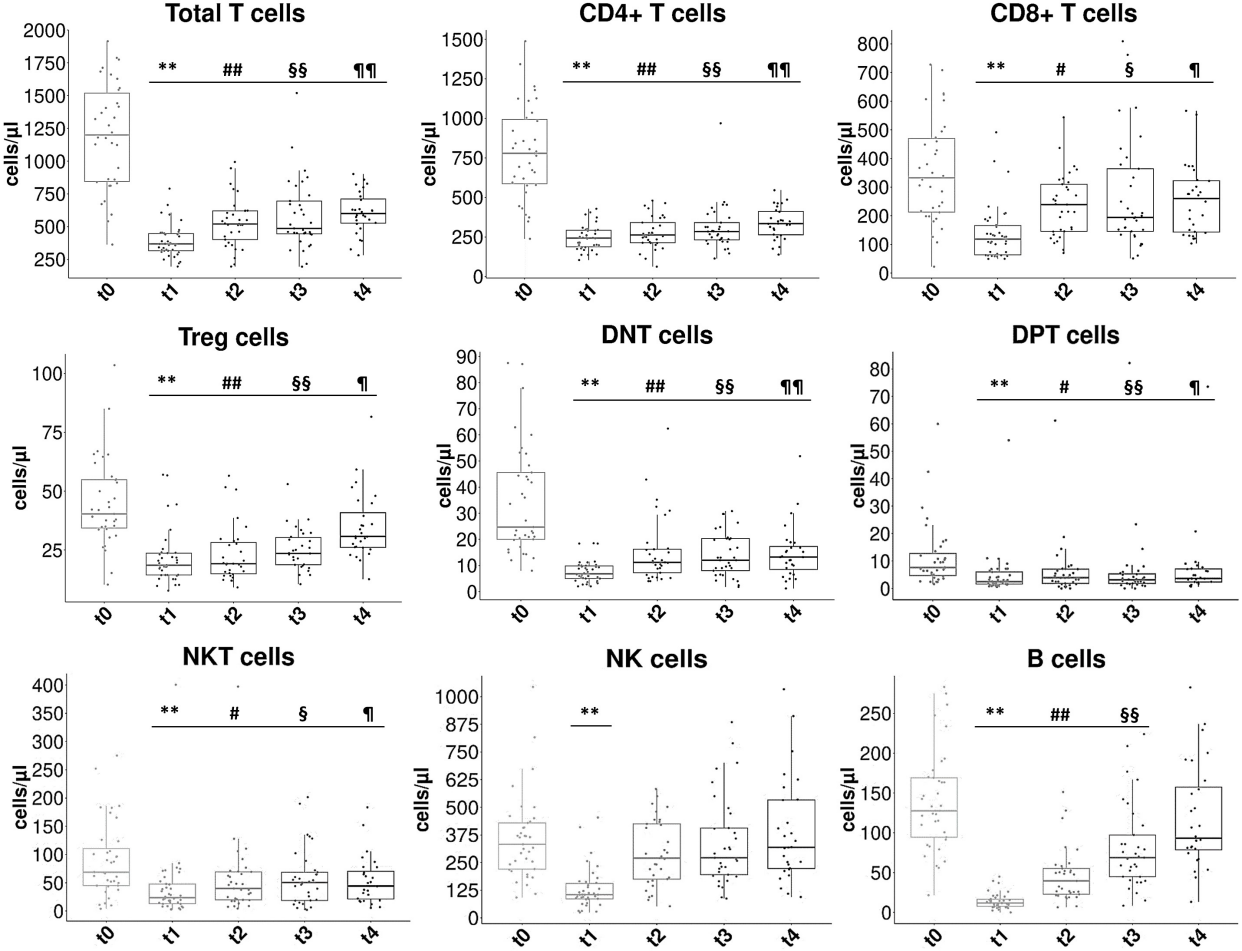
Concentration changes of lymphocyte subpopulations at the different time points considered. The box plots show the concentration values (in cells/µl) of the different peripheral lymphocyte subpopulations at the five considered time points: t0= pre-RT, t1= end of RT, t2= 3 months after RT, t3= 6 months after RT, t4= 12 months after RT. Statistical symbols (multiple comparison): **t1 vs t0 *p*<0.001; #t2 vs t0 *p*<0.05; ##t2 vs t0 *p*<0.001; §t3 vs t0 *p*< 0.05; §§t3 vs t0 *p*< 0.001; ¶t4 vs t0 *p*<0.05; ¶¶t4 vs t0 *p*<0.001.

The different peripheral lymphocyte populations considered were: total T cells (CD3+), CD4+ T cells (CD3+ CD4+ CD8-), CD8+ T cells (CD3+ CD8+ CD4-), regulatory T cells (Tregs) (CD4+ CD25+ CD127low/-), DNT cells (CD3+ CD4- CD8- CD16/CD56-), DPT cells (CD3+ CD4+ CD8+), NKT cells (CD3+ CD16/CD56+), NK cells (CD3- CD16/CD56+) and B cells (CD19+) (**Supplementary Figures 1**, **2**). The concentrations (cells/µl) of all these immune cell subtypes vary significantly (p-value<0.001) during the time points considered (**Figure 1**). In particular, they have a noticeable decrease at the end of RT (t1) compared to baseline (t0) and then tend to increase gradually during the following 12 months, without however reaching pre-RT values. Only NK cells return to initial values ​​12 months (t4) after the end of RT (t0 median: 332.5 cells/µl, IQR: 220.4-434.7; t4 median: 318.8 cells/µl, IQR: 222.9-532.9, p-value=0.848) and for B cells at time t4 the difference with the values ​​at t0 is no longer statistically significant (p-value=0.124). Total T and CD4+ T cells have the slowest recovery after RT, in fact at t4 they do not even reach 50% of the t0 values (p-value<0.001). The greatest RT toxicity is experienced by B-lymphocytes, which are reduced by approximately 90% at t1 compared to t0 (t1 median: 11.8 cells/µl, IQR: 7.6-16.5; t0 median: 127.6 cells/µl, IQR: 95.9-168.1, p-value<0.001).

**Figure 2:**
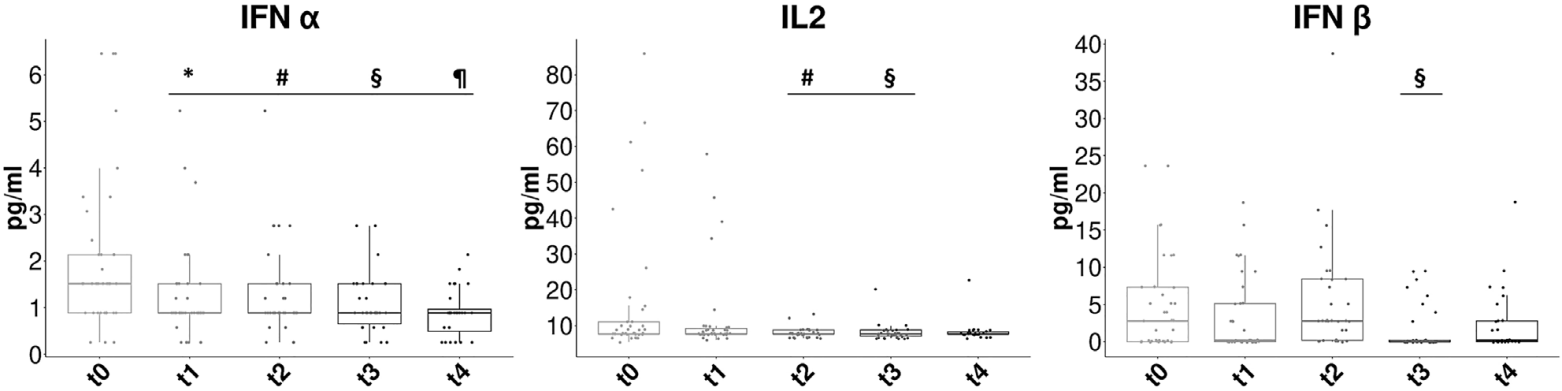
Concentration changes of cytokines at the different time points considered. The box plots show the concentration values (in pg/ml) of the serum cytokines at the five considered time points: t0= pre-RT, t1= end of RT, t2= 3 months after RT, t3= 6 months after RT, t4= 12 months after RT. We show only the value time series that have significant variation (Kruskal-Wallis *p*<0.05). Statistical symbols (multiple comparison): *t1 vs t0 *p*<0.05; #t2 vs t0 *p*<0.05; §t3 vs t0 *p*< 0.05; ¶t4 vs t0 *p*<0.05.

Regarding serum cytokines, the concentrations of IFN-α, Il-2 and IFN-β changed significantly (p-value<0.05) between the different time points considered (**Figure 2**). In particular, we found reduction of IFN-α values for all time points respect to t0 (p-value<0.05), significant reduction of IL-2 respect to t0 at t2 and t3 time points and slight subsequent increase, and IFN-β clear reduction in value at time points t3 (p-2value<0.05) and t4 (not statistically significant) respect to t0.

### Correlations between cytokines and lymphocyte subpopulations

We investigated the possible correlation between some of the cytokines tested and the lymphocyte subtypes biologically related to them (total T cells and other T cell subsets). At the end of RT, we found a negative significant correlation between CD8+ T cells and IL-2 values (p-value: 0.037, rho: -0.338). No other significant correlation was found (**Table 4**).

**Table 4:** Correlation between peripheral T lymphocyte subpopulation values and serum cytokine concentrations.

Lymphocyte subpopulation absolute value (µl^-1^)	Cytokine concentration (pg/ml)	Time point	Rank correlation (rho)	*p* value
Treg cells	IL-2	basal	-0.274	0.111
Treg cells	IL-2	End of RT	-0.283	0.088
Treg cells	IL-2	FUP 12M	-0.118	0.555
Treg cells	TGF-β1	basal	0.004	0.980
Treg cells	TGF-β1	End of RT	-0.056	0.740
Treg cells	TGF-β1	FUP 12M	0.060	0.764
Treg cells	IL-10	basal	0.209	0.227
Treg cells	IL-10	End of RT	0.022	0.894
Treg cells	IL-10	FUP 12M	0.188	0.347
Total T cells	IFN-α	basal	0.137	0.436
Total T cells	IFN-α	End of RT	-0.165	0.319
Total T cells	IFN-α	FUP 12M	0.066	0.740
CD4+ T cells	IL-2	basal	-0.063	0.718
CD4+ T cells	IL-2	End of RT	-0.292	0.075
CD4+ T cells	IL-2	FUP 12M	-0.209	0.294
CD8+ T cells	IL-2	basal	0.138	0.434
CD8+ T cells	IL-2	End of RT	-0.338	**0.037**
CD8+ T cells	IL-2	FUP 12M	-0.185	0.353
Total T cells	IL-1β	basal	0.103	0.559
Total T cells	IL-1β	End of RT	-0.141	0.395
Total T cells	IL-1β	FUP 12M	-0.332	0.089
Total T cells	IFN-β	basal	-0.279	0.110
Total T cells	IFN-β	End of RT	-0.155	0.351
Total T cells	IFN-β	FUP 12M	-0.048	0.809

FUP, follow-up; IFN, interferon; IL, interleukin; M, months; RT, radiotherapy; TNF, tumor necrosis factor; TGF, transforming growth factor. In bold, the statistically significant p-value.

### Toxicity data and correlation with TGF-β1 basal levels

In **Table 5**, we summarize the GU and GI toxicity (acute and late) associated to the RT treatment that we recorded during the follow up time of the patients.

**Table 5:** RT toxicity data of the patients.

	No symptoms	Grade 1	Grade 2	Grade 3
Genitourinary toxicity				
Acute	17 (44.7%)	14 (36.9%)	7 (18.4%)	0%
Late	19 (50%)	13 (34.2%)	4 (10.5%)	2 (5.3%)
Gastrointestinal toxicity				
Acute	16 (42.1%)	10 (26.3%)	12 (31.6%)	0%
Late	20 (52.6%)	12 (31.6%)	6 (15.8%)	0%

**Figure 3:**
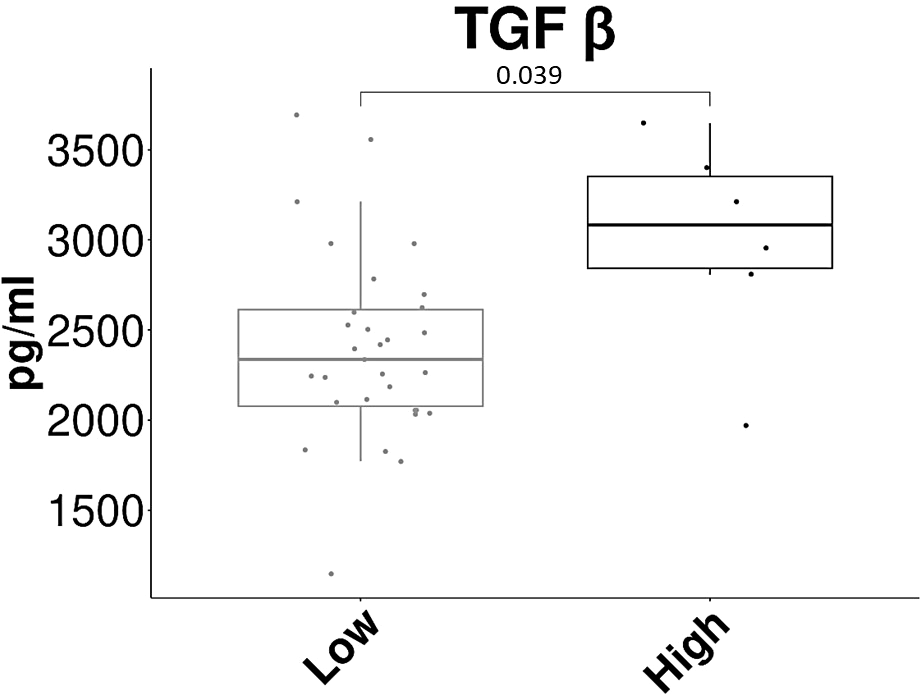
Correlation between TGF-β1 basal concentration and late genitourinary toxicity. The box plot shows the basal concentration values (in pg/ml) of serum TGF-β1 in the two groups of patients: Low= grade 0-1 of late GU toxicity, High= grade 2-3 of late GU toxicity.

We tested whether there was a correlation between the severity of late GU and GI toxicity and baseline values ​​of lymphocyte subpopulations and cytokines. In the late GU toxicity setting, we found that TGF-β1 was significantly higher in grade 2-3 (median: 3082 pg/ml, IQR: 2843-3352) than in grade 0-1 (median: 2337 pg/ml, IQR: 2076-2613) (p-value: 0.039) (**Figure 3**). We did not find the same statistically significant difference for late GI toxicity, where the values ​​for the group grade 0-1 (median: 2409 pg/ml, IQR: 2113-2843) and grade 2-3 (median: 2441 pg/ml, IQR: 2052-2601) were comparable. There wasn’t a correlation between TGF-β1 basal values and GU or GI acute toxicity grades. No other baseline parameters were significantly correlated with the higher late GU or GI toxicity grades.

### Radiation planning parameters vs clinical and biological data

Since B-lymphocytes are those cells that suffer the greatest grade of RT toxicity, we evaluated if the ratios of B cell concentrations at the concentrations at t1, t2 and t4 compared to t0 correlated with the volumes (in cm^3^) of the pelvis irradiated and with the mean dose (in Gy) of radiation received by the pelvis. We did not find any statistically significant correlation (data not shown).

We then considered whether values of pelvis irradiated volumes and pelvis RT mean dose had a correlation with the results of the IPSS questionnaire at 3 and 24 months after RT, grouping patients into mildly or moderately/severely symptomatic. Again, we found no significant correlation.

## Discussion

The immune system has recently been much studied in the field of RT. This interest is driven both by the attempt to better explain the effects of RT on immune cells and by the search for possible predictive factors of response or toxicity. In our case, we focused on the study of the peripheral variations of some parameters of the immune system in prostate cancer patients subjected to hypofractionated RT. The study of the trend of lymphocyte subpopulations following RT is present in literature in various types of cancer (1, 2, 8, 26–32).

Speaking specifically about RT for prostate cancer (PC), Eckert et al. observed, in 18 patients, that during the course of standard RT, the percentage of peripheral blood T cells, CD8+ and naïve CD4+ T cells and B cells decreased while regulatory T and NK cells increased (1).

A further study on a group of 33 PC patients found that definitive (curative intent for patients with prostate) or salvage (administered for post-prostatectomy biochemical relapse) RT cause similar effects and, in particular, that B-lymphocytes are more sensitive to both types of RT respect to T and NK cells (33).

In our previous work, we found that in PC patient’s peripheral immune cell subpopulation values decreased significantly at the end of RT, but less in the hypofractionation group than in the conventional one. Also the other smaller subgroups of T cell populations, double-negative T (DNT), double-positive T (DPT), and NKT cells significantly decreased at the end of RT with a slight tendency to recover during follow up, particularly in the hypofractionation group (8).

In a recent work of Palermo et al. (9), in accordance with our aforementioned data, total B and T cells decreased and conventional fractionated RT had a worse long-term effect on immune cells.

Another important aspect not to be underestimated is the RT cytotoxic effect on the various immune cell populations present in the lymph nodes draining the tumor which, based on the different therapeutic schemes, can be irradiated simultaneously with the tumor (34). These lymph nodes, in fact, are a very important site for the development of adaptive anti-tumor immunity.

The peculiarity of our work is that of having considered the variation in the concentration of the different peripheral blood lymphocyte subpopulations, contrary to most existing publications, which consider percentage values. In line with data from our previous work (8), we found that the concentration of B-lymphocytes is the one that undergoes the greatest decrease following RT. This data is in line with what has previously been reported in literature also for breast cancer (2, 32), gynecologic neoplasms (28) and prostate cancer (1, 29, 33). B cells slowly tend to rise during follow-up but, 12 months after RT, they do not reach the basal values. The other lymphocyte subpopulations also have the same trend, except for NK cells that are the only ones to return to pre-RT values ​​at 12 months follow up after RT. This trend is also found in other types of carcinomas treated with radiotherapy (32, 35).

As already known, RT can also have a significant effect on cytokine release (21). Some of them, such as IL-1β, IL-6, IL-8, IFN-γ, and TNF-α, have a pro-inflammatory role, others, such as IL-10 and TGF-β, an immuno-modulatory function (21).

As regards proinflammatory cytokines, many studies reported an increase in circulating levels during RT. In particular, IL-6 increases during radiotherapy for PC and is also associated with acute genitourinary toxicity (4). In another study (36), IL-6 and also IFN-gamma increased during prostate intensity-modulated radiation therapy (IMRT) and IL-2 and IL-1 levels were correlated to GI and GU toxicity.

In a study of 18 PC patients, Singh J et al. found an association, not statistically significant, between TNF-α, IL-6 and TGF-β1 levels and the severity of GU and GI toxicity (5).

In a group of 39 localized or locally advanced PC patients undergoing RT, Kopcalic K et al. found that circulating levels of IL-6 correlated with fatigue and TGF-β1 with GU toxicity (37).

Regarding the analysis of cytokines in our work, we found a significantly different basal concentration of IFN-γ between definitive and post-operative patients. The correlation between the presence of the prostate and the concentration of IFN-γ was reported also in the study of Stanojkovic et al. (4). In their study, however, the concentration was higher in the definitive group than in the post-operative one. The same authors identified a correlation between higher concentrations of IL-6 and higher degrees of acute GU toxicity. As far as we are concerned, we did not find any correlation between serum IL-6 levels, basal or post-RT, and the other variables considered. Among all 10 cytokines analyzed, we found an interesting correlation between TGF-β1 basal serum values and late GU toxicity, an insidious RT effect feared by clinicians. In various works, on the prostate as well as on lung or breast cancer (15, 38, 39), the correlation between TGF-β and RT toxicity has been studied. In particular, in PC patients, Kopcalic et al. found a statistically significant correlation (*p*=0.036) between pre-treatment serum TGF-β1 concentration and the higher grades of acute GU toxicity (37). In locally advanced lung cancer, it was observed that at the time of 30-48 Gy irradiation, higher changes of serum TGF-β compared to baseline were correlated with higher degrees of radiation-induced lung injury (38). In stage 0-1 breast cancer patients, the mean TGF-β1 serum levels pre-accelerated hypofractionated partial breast irradiation correlate with the development of moderate to severe radiation-induced fibrosis (39). In our group of PC patients, serum TGF-β1 median concentration was significantly higher in patients who developed grades 2-3 of late GU toxicity than in those with grades 0-1.

One of our limitations is the small size of our group of patients, especially the 12-month time point for which we were only able to collect samples from 28 patients. This could probably also influence the non-statistically significant variation over time for many of the cytokines considered.

As regards the analysis of the effects of hypofractionated RT on the immune system in PC, this work longitudinally analyzes in detail the peripheral concentration changes of the different populations of immune cells within a year from the end of radiotherapy. We show which populations suffer the greatest toxicity (B cells), which recover the slowest (total T and CD4+ T cells) and in any case does not reach baseline levels at one year and which, one year after RT, manages to return to initial values ​​(NK cells). Furthermore, very important information for clinicians, we highlight how baseline serum values ​​of the cytokine TGF-β1 can be a potential predictive marker of severe late genitourinary toxicity.

## Data Availability

The raw data supporting the conclusions of this article will be made available by the authors, without undue reservation.
