# Supplementary figures and images for: Hypofractionated radiotherapy with simultaneous integrated boost for localized prostate cancer patients: effects on immune system and prediction of toxicity
Source: Front Immunol. 2024 Oct 28;15:1457839. doi: 10.3389/fimmu.2024.1457839 (PMC11550950; doi:10.3389/fimmu.2024.1457839)

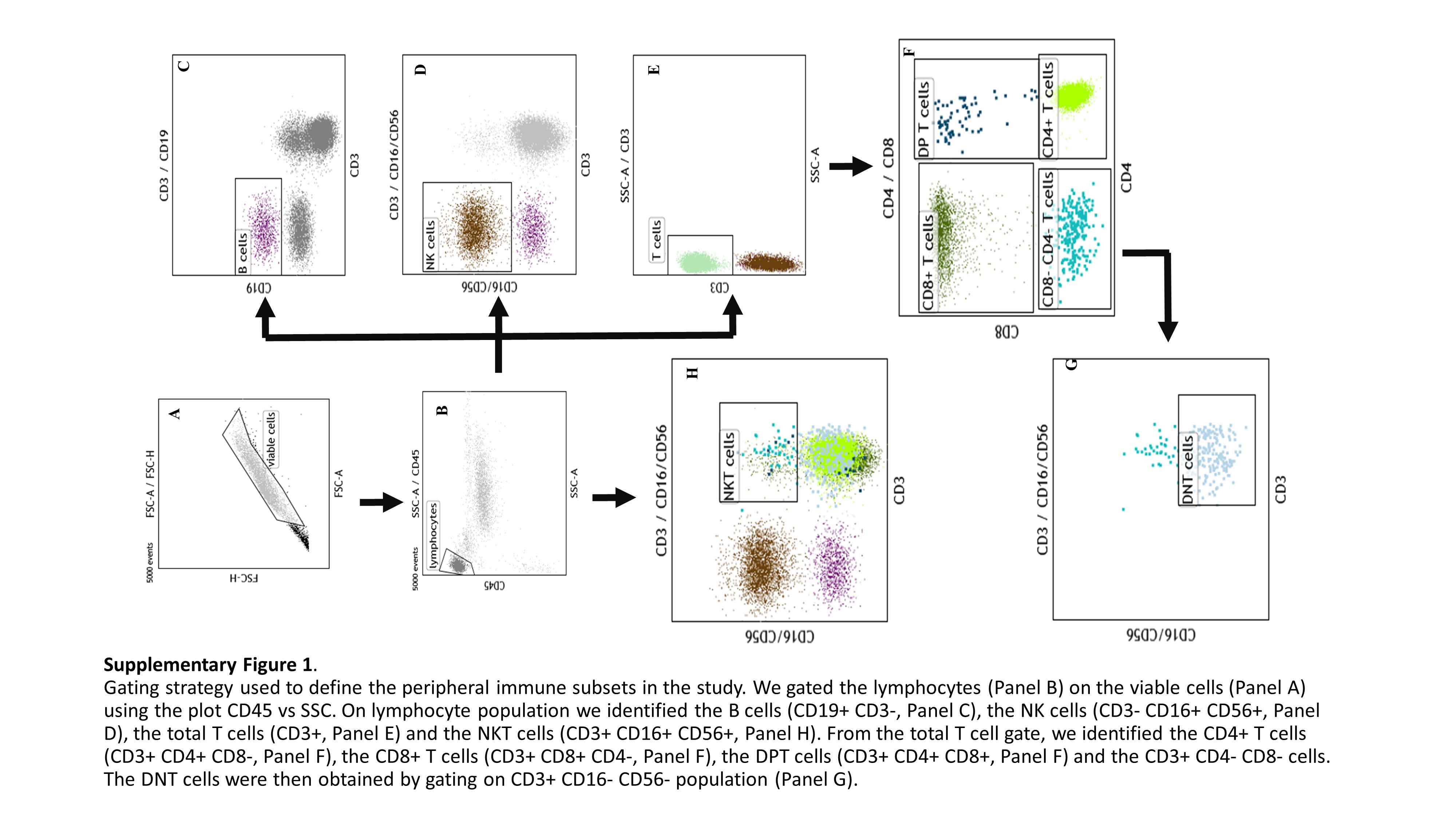

Supplement: Supplementary file 1 [file Image1.jpg]

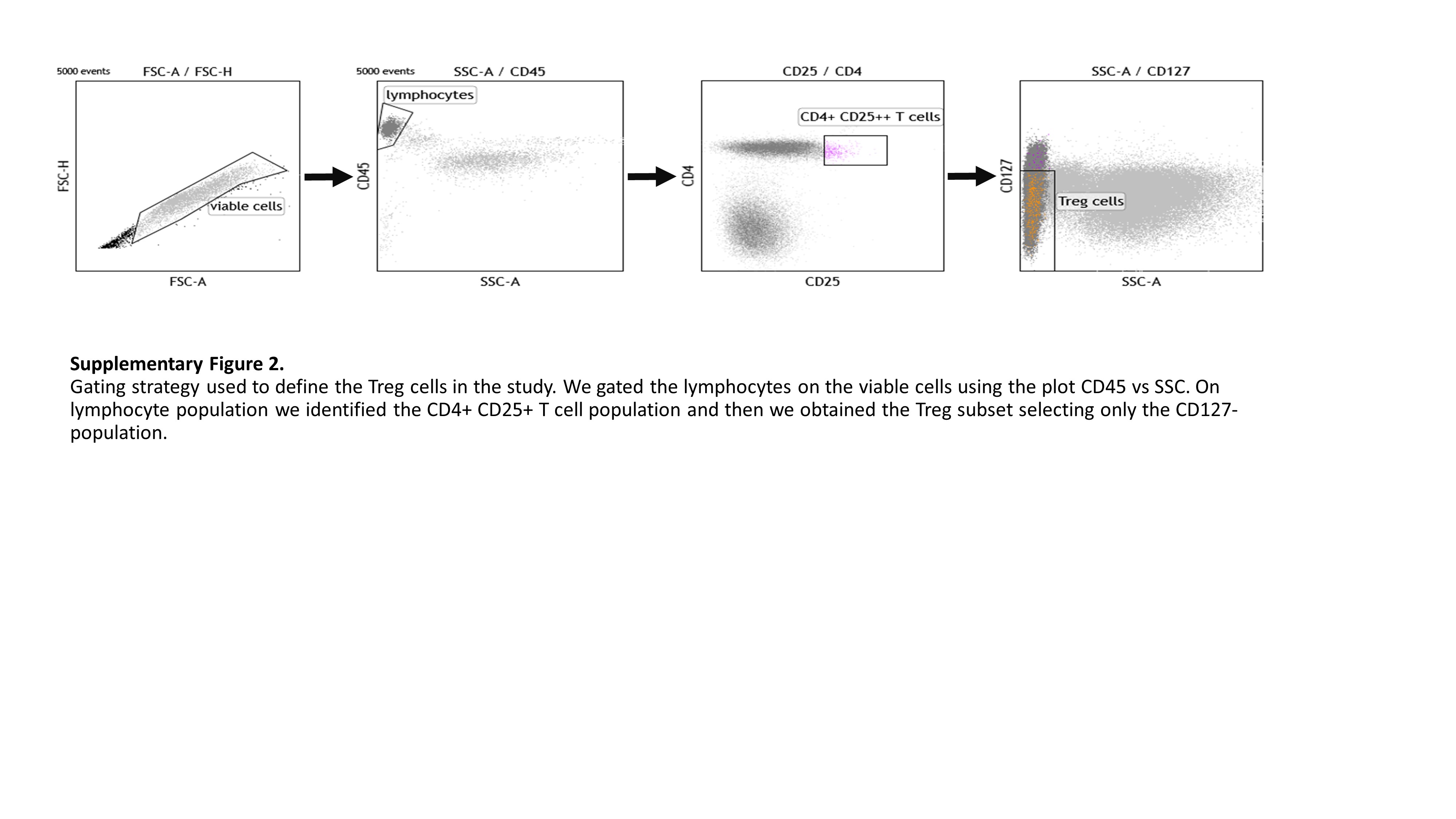

Supplement: Supplementary file 2 [file Image2.jpg]
